# Blocking Mimicry Makes True and False Smiles Look the Same

**DOI:** 10.1371/journal.pone.0090876

**Published:** 2014-03-26

**Authors:** Magdalena Rychlowska, Elena Cañadas, Adrienne Wood, Eva G. Krumhuber, Agneta Fischer, Paula M. Niedenthal

**Affiliations:** 1 Department of Psychology, University of Wisconsin-Madison, Madison, Wisconsin, United States of America; 2 Université Blaise Pascal, Clermont-Ferrand, France; 3 Institut de Psychologie du Travail et des Organisations, Université de Neuchâtel, Neuchâtel, Switzerland; 4 Division of Psychology and Language Sciences, University College London, London, United Kingdom; 5 Department of Psychology, University of Amsterdam, Amsterdam, the Netherlands; UCLA, United States of America

## Abstract

Recent research suggests that facial mimicry underlies accurate interpretation of subtle facial expressions. In three experiments, we manipulated mimicry and tested its role in judgments of the genuineness of true and false smiles. Experiment 1 used facial EMG to show that a new mouthguard technique for blocking mimicry modifies both the amount and the time course of facial reactions. In Experiments 2 and 3, participants rated true and false smiles either while wearing mouthguards or when allowed to freely mimic the smiles with or without additional distraction, namely holding a squeeze ball or wearing a finger-cuff heart rate monitor. Results showed that blocking mimicry compromised the decoding of true and false smiles such that they were judged as equally genuine. Together the experiments highlight the role of facial mimicry in judging subtle meanings of facial expressions.

## Introduction

Accurate judgment of other people's facial expressions is critical in everyday social interactions. Recent theories suggest that such judgments are sometimes subtended by automatic facial mimicry, defined as overt or covert imitation of perceived expression [1], [2], [3]. The claim is that automatic facial mimicry helps a perceiver internally simulate and re-experience an emotion that corresponds to the perceived expression, thereby aiding in processes of recognition and interpretation [4], [2], [5]. This “embodiment” hypothesis derives from theories that hold that perception and action are tightly coupled, such that simulating a perceived action enables its perceptual encoding [6], [7], [8]. The hypothesis has been supported by a handful of studies on the decoding of facial expression. For example, Oberman and colleagues [3] blocked mimicry on the lower half of perceivers' faces and observed poorer recognition of happiness and disgust expressions, but no difference for sadness or fear. Ponari, Conson, D'Amico, Grossi, and Trojano [9] replicated the findings for happiness and disgust, and further demonstrated that blocking mimicry of the upper face resulted in poorer recognition of anger. These results are impressive because participants of the experiments viewed and classified facial expressions that were prototypic, and thus easily categorized. In theory, people may be most served by embodied simulation when they are both highly motivated to understand the perceived expression and when the expression itself is non-prototypic or conveys nuanced meanings [2], [10].

A smile is a good example of a nuanced facial expression. Human smiles can communicate not only happiness [11], [12], but also other emotions and motivations [13]. An accurate judgment of these motives may therefore be more dependent on facial mimicry, making smiles ideal expressions for studying mimicry. Spontaneous smiles that reflect feelings of enjoyment – so-called *true* smiles – are a particularly well-defined class [14]. Such smiles elicit pleasure in the perceiver and thereby can act as powerful social rewards [15], triggering positive emotion [16] and cooperative behavior [17]. *False* or *polite* smiles are less rewarding and are displayed when people want to mask unpleasant feelings or show positive affect they do not actually feel [18]. The distinction between true and false smiles involves not only the action of certain facial muscles (such as the *cheek raiser*, action unit (AU) 6, in Facial Action Coding System, FACS, [19] but also subtle dynamic properties such as the synchrony of different facial actions [20], [21]; the time course of the expression's onset, apex, and offset [22]; and the amount of eye constriction [18], [23]. Judging smile genuineness is a complex task that requires simultaneous integration of these features. Consequently, it is likely to be supported by embodied responses such as facial mimicry. It is also worth noting that facial expressions of happiness are especially appropriate for studying facial mimicry because their imitation elicits high levels of muscle activity and is easy to detect [3].

The goal of the present research was to provide a critical test of the role facial mimicry plays in the judgments of smile authenticity. In the first experiment reported here, we introduce and test a novel mimicry inhibition technique. We then employ the technique in the two following experiments to clarify the role that mimicry plays in distinguishing between true and false smiles.

Our experiments improve on and extend initial evidence for the role of mimicry in decoding true and false smiles reported by Maringer, Krumhuber, Fischer, and Niedenthal [24]. In that work, Maringer and colleagues showed videos of animated agents expressing empirically validated “true” and “false” dynamic smiles [25] to their participants. Half of the participants were able to freely mimic the smiles, whereas the remaining half held pens in their mouth such that facial mimicry was functionally blocked. Participants' task was to rate the genuineness of each smile. Findings revealed that participants in the mimicry condition judged true smiles as more genuine than false smiles, consistent with validation studies. However, in the mimicry-blocked condition, participants' judgments of genuineness did not vary by smile type. Instead, all smiles were rated as equally genuine. This result was consistent with the hypothesis that the ability to mimic smiles is essential for distinguishing among their subtle meanings.

The study by Maringer and colleagues [24] represented the first step in demonstrating how facial mimicry supports perceivers' detection of subtle differences between smiles, but it was not without its limitations. The stimuli used were synthetic faces expressing “true” and “false” smiles, with true smiles defined as having a slower onset and a briefer apex compared to the false smiles [25]. While such stimuli are valuable because they have been precisely constructed and controlled, they do lack external validity and cannot represent a situation in which motivations to express true and false smiles are present. Whenever possible, it is important that research compares the mechanisms involved in the decoding of synthetic and real human facial expressions.

Another potential limitation of the study by Maringer et al. [24] is the lack of control conditions to support a strong causal conclusion about the role of facial mimicry in decoding smiles. As mentioned, half of the participants completed the experimental task without any interfering activity (free mimicry condition) and the other half held a pen sideways between their lips and teeth, exerting only slight pressure (mimicry-blocked condition). Because holding the pen in the mouth requires some sustained attention, it is possible that the findings of the study, specifically that blocking mimicry compromised decoding accuracy, were due to distraction caused by the method for blocking mimicry. Perhaps the participants with the pen were simply sloppier in their judgments of genuineness.

Finally, Maringer and colleagues did not measure the effects of the pen-in-the-mouth manipulation on facial mimicry. Their manipulation elicits less interference with mimicry than a similar paradigm that has also been described in the literature (i.e., holding a pen between the teeth, without touching it with the lips [3], [9], [26]. Since Maringer and colleagues [24] did not report empirical evidence for the effectiveness of their manipulation of facial mimicry, it is impossible to draw strong conclusions from their findings about the role of mimicry in the decoding of smiles. Finally, the between-subject design employed by the researchers does not allow taking into account important individual differences in both participants' tendency to mimic and the effectiveness of mimicry-blocking manipulation.

In order to address these shortcomings found in previous work, the present research employed a number of strategies allowing to ground stronger conclusions about the role of facial mimicry in decoding smiles. First, we used rich, naturalistic stimuli representing spontaneous true and posed false smiles. Specifically, participants saw video recordings of real human participants smiling in response to real, amusing (versus neutral) stimuli.

In Experiment 2, in order to control for the possibility that blocking facial mimicry distracts participants resulting in poor decoding of smiles, we added a control condition to free-mimicry and mimicry-blocked conditions. In this third condition participants held a squeeze ball (“stress ball”) in their non-dominant hand as they performed the smile decoding task. They were thus free to mimic the stimuli, but, like participants in the mimicry-blocked condition, they had an additional, potentially distracting task to perform. In Experiment 3 we implemented further control by adding distraction to the free mimicry condition itself. In that condition, participants wore a finger-cuff heart rate monitor such that they experienced the same amount of experimental involvement as participants in the other conditions. If the mimicry-blocked participants in the Maringer et al. study were less accurate in decoding true and false smiles because they were distracted by the pen-in-the-mouth manipulation, then the participants holding a squeeze ball or wearing a finger cuff in the present studies should also be less accurate in decoding smiles.

Finally, in this research we introduce and validate (Experiment 1) a new procedure for inhibiting mimicry, namely the wearing of a plastic mouthguard. This device is then used in Experiments 2 and 3. Mouthguards are used in contact sports, such as football and boxing, in order to prevent injury to the teeth, jaw, and mouth [27]. They are made of thermo-plastic materials and are individually shaped to the mouth so that they fit closely around the wearer's teeth. When inserted, the mouthguard slightly stretches the mouth and cheeks, keeps the mouth in a stable position, and reduces facial movements without requiring the active attention of the wearer. Thus, mouthguards should effectively inhibit or at least disrupt the dynamics of facial mimicry. Anecdotal evidence corroborates this claim: athletes report that they strategically remove the guard when mobilizing emotional behavior. In Experiment 1 we measured facial muscle activity with and without a mouthguard in order to test the effectiveness of this technique for blocking facial mimicry.

To summarize, in the three experiments reported here we introduce and test the efficacy of a mouthguard technique for blocking facial mimicry (Experiment 1), and then use the procedure in two experiments that test the role of facial mimicry in decoding true and false smiles. Participants in Experiments 2 and 3 saw dynamic human true and false smiles and rated them on scales of genuineness. We expected participants in mimicry-blocked conditions to show poorer accuracy in discriminating between the two types of smiles compared to the participants in other conditions, able to freely mimic the stimuli. Taken together, the three experiments presented here provide strong evidence in support of the prediction that facial mimicry plays a functional role in the processing of smile meaning.

## Experiment 1

In order to investigate the efficacy of mouthguards as mimicry inhibitors, in Experiment 1 we compared the facial muscle activity of participants with and without “boil and bite” mouthguards as they viewed videos of true and false smiles.

### Method

*All reported experiments were conducted according to the appropriate ethical guidelines and approved by the Conseil Restreint, a department-wide ethics committee at Blaise Pascal University. All participants were at least 18 years old. All of them provided written informed consent to take part in the three experiments reported in the manuscript. We analyzed only anonymous data.*

#### Participants and design

Forty-two students (5 men, 37 women, age *M* = 19.12 years, *SD* = 1.47) at Blaise Pascal University, France, took part in the experiment and were paid €10. All participants were at least 18 years old. Eight participants (7 female) were not French and their responses were excluded from further analyses because of the possibility that facial behavior varies across cultures [28]. We also dropped data from one female participant because of the large number of trials preceded by intense facial activity (it is worth noting that removing those participants did not have a significant impact on the observed patterns of results – significance tests can be obtained upon request). Participants watched 12 videos of true and false smiles while wearing a mouthguard and under conditions of free mimicry. Thus, the experiment followed a 2 (Smile Type: true, false) by 2 (Mimicry Condition: free, blocked) within-subject design, where mimicry conditions were counterbalanced across participants. This and all other experiments reported in the present article were conducted according to the appropriate ethical guidelines and approved by the Conseil Restreint, a department-wide ethics committee at Blaise Pascal University.

#### Stimuli

We used six videos of true smiles and six videos of false smiles, selected from stimuli developed and described in [29]. Films started and ended with a neutral expression and were extracted from recordings of participants (4 males and 2 females) performing an experimental task [29]. True smiles were spontaneous reactions to amusing stimuli accompanied by self-reported high positive emotions (i.e., pleasure, amusement, and happiness ratings of 3 or higher on a 7-point scale ranging from 1-*not at all* to 7-*extremely*), whereas false smiles represented deliberate actions of participants asked to look as if they felt amused (and were accompanied by reported low or no positive emotions, i.e., pleasure, amusement, and happiness ratings of 2 or lower). All smiles were of moderate intensity. Facial activity in every video was scored by two FACS-trained coders. True smiles (*M* = 3.50 s, *SD* = 1.05) included both AU 12 (lip corner puller) and AU 6 (cheek raiser), whereas false smiles (*M* = 2.50 s, *SD* = 0.55) included only AU 12. False smiles were also coded as more asymmetric compared to true smiles. Perceivers' ratings [29] were consistent with these objective differences: observers judged false smiles as significantly less amused and less genuine than true smiles. All smiles were displayed as movie clips (1368×1026 pixels, 25 frames/s) in E-Prime Version 2.0 (Psychology Software Tools) and shown in random order.

#### Procedure

Participants first provided written informed consent to take part in the study. They worked individually, seated in front of a 14″ screen connected to a PC. As they viewed videos of true and false smiles, we recorded the EMG activity of participants' zygomaticus major, the main muscle involved in smiling. Videos were displayed on a black screen, separated by self-paced pauses (no less than 500 ms). Given that the technique of EMG requires multiple repetitions of the same stimulus [30], [31], each of the 12 sequences was presented three times, for a total of 72 trials presented in two randomized blocks (36 in the free mimicry and 36 in the blocked mimicry condition). The order of conditions was counterbalanced across participants. Before fitting and inserting the mouthguard, participants learned that our goal was to stabilize their facial muscles because their activity could interfere with the experimental task. Then, each participant received a new, transparent “boil and bite” mouthguard, still in the unopened box. We provided hot and cold water, along with the instructions on how to properly mold the mouthguard using tongue and biting pressure.

Electrical activity of the zygomaticus major was recorded on the left side of the face, consistent with established guidelines [32], using bipolar 10 mm Ag/AgCL surface electrodes. We measured the EMG raw signal with a 16 Channel Bio Amp amplifier (ADInstruments, Inc.). The signal was then digitized by a 16 bit analogue-to-digital converter (PowerLab 16/30, ADInstruments, Inc.), and stored with a sampling rate of 1000 Hz.

#### Data preprocessing

EMG recordings were preprocessed using LabChart 7 (ADInstruments, Inc.). Recordings were filtered with a 10-Hz high-pass filter, a 400-Hz low-pass filter, and a 50-Hz notch filter, and segmented from 500 ms before to 2 seconds after the video onset, given that the most distinct facial reactions occur during the first second after stimulus onset [33], [34]. In order to control for random facial movements prior to the stimulus onset, we excluded from further analysis trials on which the z-scores of mean amplitude of the baseline (500 ms before the stimulus onset) were higher than 3 (on average 1 out of 72 trials per participant, never more than 3). The remaining data were then expressed as percentages of the baseline and averaged per condition in 20 time bins of 100 ms, in order to reflect how the EMG signal evolved after the onset of true and false smile videos.

### Results

Statistical analyses were performed using PASW Statistics 18 (SPSS, Inc., Chicago, IL) and RStudio (version 0.96.331, RStudio, Inc.).

#### Overall effect of condition on EMG responses

Given that the mouthguard stretches the mouth and the cheeks, we did not expect it to completely inhibit facial movements but rather to induce irrelevant muscle activity that would interfere with participants' mimicry. To test this hypothesis, we examined how average responses of zygomaticus major in the first 2 seconds after the video onset varied as a function of smile type (true, false) and mimicry condition (free, blocked). Data screening and Shapiro-Wilk tests revealed that the dataset violated normality assumptions (see Table 1 for details). A Wilcoxon Signed-ranks test indicated that when participants could freely mimic the video stimuli, they imitated true smiles to a greater extent (*M* = 1.336, *SD* = 1.476) than false smiles (*M* = 1.08, *SD* = .27), *Z* = −2.64, *p* = .008, consistent with previous research [16], [35]. This difference disappeared when participants were wearing a mouthguard (respectively, *M* = 1.08, *SD* = .16, *M* = 1.06, *SD* = .14), *Z* = 0.12, *p* = .908.

**Table 1:** Responses of Zygomaticus Major as a Function of Mimicry (free, blocked) and Smile Type (true, false) in Experiment 1.

Mimicry	Free		Blocked	
Smile Type	True	False	True	False
*M*	1.336	1.085	1.081	1.062
*SD*	1.476	.267	.159	.143
*S-W* (*df* = 33)	.261	.681	.783	.927
Skewness	5.604	2.560	2.559	.807
Kurtosis	31.876	6.989	10.220	.409
*p*	.000	.000	.000	.029

*Note.* EMG scores are expressed as percentages of baseline (500 ms before the stimulus onset).

#### Mapping EMG data on stimuli's facial activity

In order to assess the time course of participants' zygomaticus major activity in both conditions, we compared their EMG responses to the smile dynamics of the stimuli videos, extracted with the Computer Expression Recognition Toolbox – CERT [36].

#### *The Computer Expression Recognition Toolbox*

CERT is a software tool for automatic facial expression recognition, trained to code 19 FACS action units as well as prototypic facial expressions, facial features, and head orientation. It is a useful alternative to human FACS coding because it allows for quick frame-by-frame coding of videos of facial expressions. More precisely, CERT outputs can describe a given facial expression as series of numbers corresponding to the intensity of each facial action unit for each video frame. Intensities are described as distances between the values of each facial unit detected in the source video and the support vector machines classifying this particular facial unit [36]. Preliminary empirical evidence suggests that CERT outputs are correlated with the EMG activity of the muscles supporting the corresponding action units [36], [37]. CERT is especially useful for research on smiles, because it not only detects AU 12 (lip corner puller), but is also equipped with a separate smile detector that significantly correlates with human judgments of smile intensity [38].

We used CERT to explore patterns of participants' mimicry of true and false smiles in the conditions of free and blocked mimicry. We defined facial mimicry in terms of positive correlations between the intensities of smiles detected by CERT in the video stimuli and the EMG recordings of participants' zygomaticus major. If wearing a mouthguard interferes with facial mimicry, positive correlations between the CERT output and EMG recordings should not be observed.

#### *Analyses*

To test these predictions, we compared CERT outputs for smile detection and AU 12 during the first 2000 ms after stimulus onset with participants' zygomatic activity recorded for the same time period under the conditions of free and blocked mimicry. CERT distances and EMG activations were expressed as z-scores and correlated using the nonparametric Spearman's rank order correlation coefficient (i.e., Spearman's rho).

In the condition of free mimicry, Spearman's rho revealed large [39] positive relationships between AU 12 detected in the video stimuli and the participants' zygomaticus activity. The correlations were significant for true and false smiles, respectively, *rs* (18) = .67, *p* = .001; *rs* (18) = .79, *p*<.001, suggesting that both types of stimuli elicited facial mimicry. We observed a similar pattern when zygomaticus activity in reaction to true and false smiles was correlated with the outputs of the smile detector, respectively *rs* (18) = .57, *p* = .009; *rs* (18) = .81, *p*<.001. Using the standard Fisher's z-transformation and subsequent comparison of Spearman coefficients [40] did not reveal significant differences in the degree of participant-target synchrony for genuine and false smiles (*z* = −0.75, *p* = .23 for AU 12; *z* = −1.38, *p* = .084 for the smile detector).

Importantly, when participants were wearing a mouthguard, their facial responses did not correlate with the CERT codings of the smile stimuli, suggesting that participants imitated neither the true (*rs* (18) = .22, *p* = .346 for AU 12; *rs* (18) = .11, *p* = .654 for smile detector) nor the false smiles (*rs* (18) = −.23, *p* = .336 for AU 12; *rs* (18) = −.23, *p* = .326 for smile detector).

In summary, results of the two analyses reported show that participants imitated smiles that they viewed when they were allowed to mimic freely. More importantly, we also show that wearing a mouthguard decreases both the amount of mimicry and the degree to which participants' facial expressions corresponded to those in the videos, compared to the condition without mouthguard. We can thus conclude that using this device is a valid procedure for interfering with facial mimicry.

## Experiment 2

The goal of Experiment 2 was to test whether mouthguards alter participants' ratings of the genuineness of smiles used in Experiment 1. Support for this prediction would suggest that the ability to mimic smiles moderates processing of subtle differences in the meaning of facial expression. Furthermore, in order to rule out potential alternative interpretations of the effect of the mouthguard on participants' ratings, we included an appropriate control condition.

### Method

#### Participants and Design

Seventy-eight undergraduate students (10 men, 68 women, age *M* = 20.09 years, *SD* = 2.45) at Blaise Pascal University, France, participated in exchange for course credit. All participants were at least 18 years old. They were randomly assigned to the conditions of a 2 (Smile Type: true, false) by 3 (Mimicry Condition: free, blocked, muscle-control) factorial design, where the first factor varied within subjects and the second varied between subjects. Each participant was tested individually.

#### Procedure

As in Maringer et al. [24], the pretext for the research was the development of a collaborative system in which people could attend meetings and conferences online. After providing their written consent, participants read specific instructions stating that our goal was to evaluate features of sample facial expressions that would be displayed on the computer screen. Participants were then randomly assigned to one of the three mimicry conditions, and rated each face according to how genuine the expressed smile was on 5-point scales, where 1 meant that the smile was not at all genuine and 5 meant that the smile was very genuine. Each participant saw all 12 videos from Experiment 1 one time each.

In the *free* mimicry condition no additional information was provided. Participants in the *blocked* mimicry condition were informed that past research had shown that individuals' extraneous bodily movements interfered with the performance of the task, and that it was important that some of their muscles be otherwise occupied. Similarly to Experiment 1, subjects were told that their face muscles would be stabilized throughout the experiment by a sports mouthguard. Each participant received then a new transparent mouthguard, along with hot and cold water and instructions on how to mold the mouthguard to fit the mouth and teeth snuggly.

Participants in the *muscle*-*control* condition heard the same information about extraneous bodily movements, but they received a small “stress ball” about 7 cm in diameter, which they were instructed to hold firmly in their non-dominant hand throughout the experiment. This condition thus controlled for the potential distracting aspects of the mouthguard used in the blocked mimicry condition.

Upon completion of the task, the experimenter debriefed the subjects. Participants in the blocked condition could keep their mouthguards.

### Results

Average genuineness ratings were submitted to an ANOVA with one within-subjects factor (Smile Type: true, false) and one between-subjects factor (Mimicry: free, blocked, control). Data for one participant were not properly recorded and were thus eliminated from final analyses.

**Figure 1:**
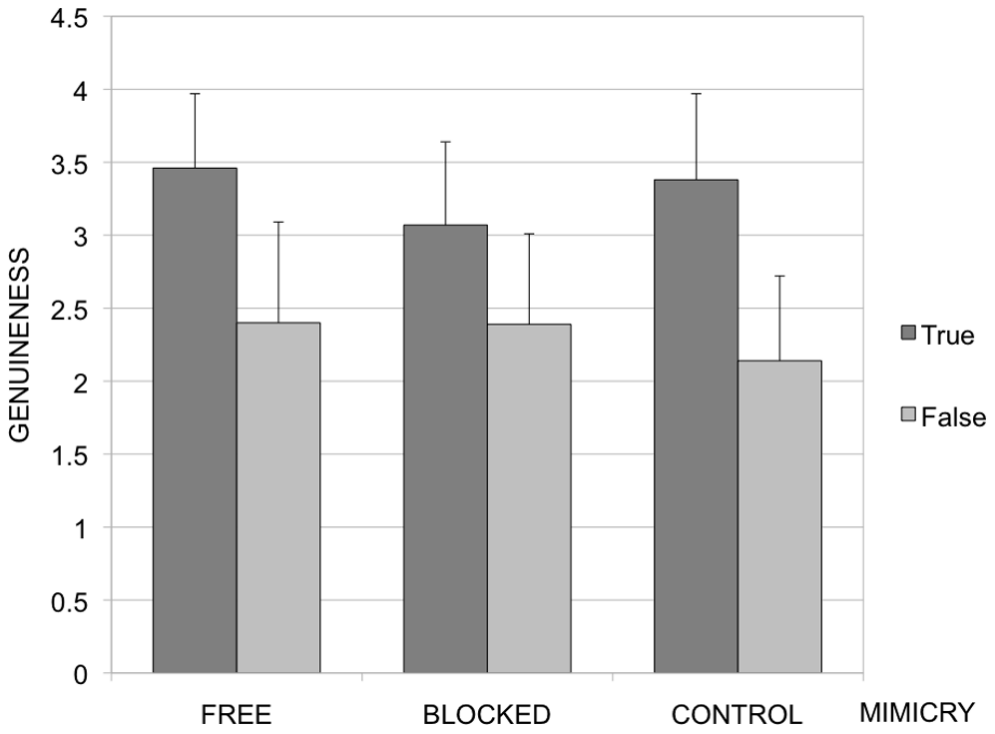
Genuineness ratings of true and false smiles in the free, blocked and muscle-control (squeeze ball) condition of Experiment 2. Error bars represent standard errors.

A main effect of Smile Type was observed, *F*(1,74) = 185.86, *p*<.001, η^2^ = 0.72 with true smiles rated as more genuine (*M* = 3.31; *SD* = .56) than false smiles (*M* = 2.31; *SD* = .64), see Figure 1 for details. More importantly, we also observed a significant interaction between Smile Type and Mimicry, *F*(2, 74) = 5.98, *p* = .004, η^2^ = 0.14, showing that participants assigned to the free-mimicry and the muscle-control conditions distinguished more between true and false smiles in their ratings of genuineness than did participants in the blocked-mimicry condition (see Figure 1). Specific comparisons revealed that the difference between the free mimicry and muscle-control condition was not significant, *F*(1, 49)<1, while the differences between free and blocked, and muscle-control and blocked conditions were significant *F*(1, 49) = 5.60, *p*<.022, η^2^ = 0.10, and *F*(1, 50) = 10.34, *p* = .002, η^2^ = 0.17, respectively.

Thus, Experiment 2 supported the prediction that participants allowed to mimic freely, with or without a distracting task, would differentiate more in their genuineness ratings of true and false smiles compared to participants whose mimicry was blocked with a mouthguard.

## Experiment 3

This study was conducted in order to replicate Experiment 2 and to further refine the comparison between the free mimicry and muscle-control conditions. We wanted to ensure that the reduced discrimination between true and false smiles in the mouthguard condition was truly due to a reduction in facial mimicry, and not because the mouthguard was distracting or heightened self-consciousness. Therefore, in Experiment 3, we modified the free mimicry condition to involve specific instructions and additional materials so that it better matched the procedures in the “stress ball” and mouthguard conditions and was equally distracting for participants. Participants in this new “free mimicry” condition were fitted with a finger heart rate monitor and informed that their heart rate would be measured during the task. The heart rate monitor is comparable to the mouthguard as it requires initial fitting, makes participants similarly aware of their bodies, and presumably has a similar effect on attention throughout the task.

### Method

#### Participants and design

Sixty-six undergraduate students (9 men, 57 women, age *M* = 20.46 years, *SD* = 6.31) at Blaise Pascal University, France participated in exchange for course credit. All of them were at least 18 years old. None of them had participated in Experiment 2. Participants were randomly assigned to the conditions of a 2 (Smile Type: true, false) by 3 (Mimicry: free, blocked, and muscle-control) factorial design as in Experiment 2.

#### Stimuli and procedure

All participants provided written informed consent to take part in the study. The stimuli and procedure largely replicated Experiment 2, with the exception of several small changes made to the instructions and materials used in the free mimicry condition. For this condition, participants were informed that past research had shown that some physiological responses were related to the performance of this task, and so, it was important for us to measure their heart rate. A heart rate monitor was then secured to the index finger of their non-dominant hand for the duration of the experiment. The monitor did not record any data and was only used to control for participants' potential distraction.

### Results and Discussion

**Figure 2:**
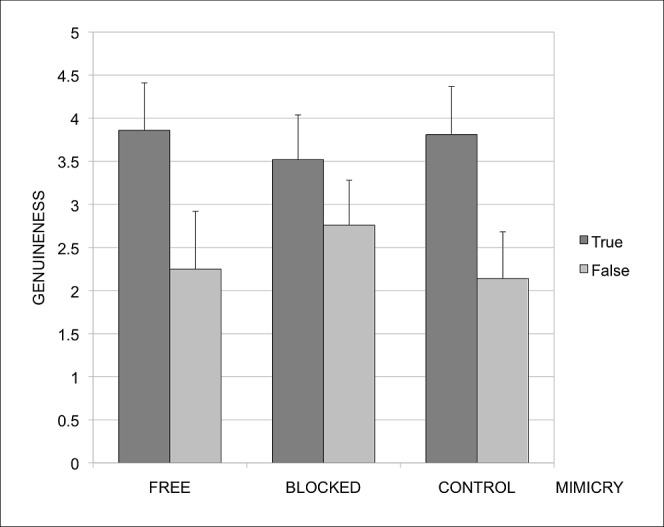
Genuineness ratings of true and false smiles in the free (finger cuff), blocked and muscle-control (squeeze ball) condition of Experiment 3. Error bars represent standard errors.

As before, genuineness ratings were submitted to an ANOVA with one within-subjects factor (Smile Type: true, false) and one between-subjects factor (Mimicry: free, blocked and muscle-control). A main effect of Smile Type was observed, *F*(1, 63) = 338.61, *p*<.001, η^2^ = 0.84, with true smiles rated as more genuine (*M* = 3.73; *SD* = .56) than false smiles (*M* = 2.34; *SD* = .65), see Figure 2 for details. More importantly, we also found a significant Mimicry by Smile Type interaction, *F*(2, 63) = 17.24, *p*<. 001, η^2^ = 0.35, such that participants assigned to free mimicry and muscle-control conditions discriminated more in their ratings of genuineness between true and false smiles (see Figure 2). The differences between free mimicry and muscle-control conditions were not significant, *F*(1, 43)<1, while differences between the free mimicry and blocked mimicry conditions, and between the muscle-control and blocked mimicry conditions were highly significant *F*(1, 42) = 24.59, *p*<.001, η^2^ = 0.40, and *F*(1, 41) = 30.40, *p*<.001, η^2^ = 0.43, respectively. Experiment 3 thus constituted a successful replication of the second experiment. It also better controlled for potential confounds in the mimicry and control conditions, showing that being able to freely mimic the perceived smiles supported participants' accuracy in judgments of authenticity, even when the participants were potentially distracted by other manipulations.

## General Discussion

The present research was conducted in order to provide a careful test of the role of facial mimicry in the decoding of smiles. The first study validated the use of a mouthguard as an effective inhibitor of facial mimicry. Having participants wear a mouthguard was shown, in Experiment 1, to disrupt the mimicry response to the perceived smiles, such that participants' EMG activity did not reflect the amount of smiling in the video stimuli. In Experiments 2 and 3 we tested the hypothesis that inhibiting facial mimicry with the mouthguard resulted in poorer decoding of true and false smiles. Unlike previous tests of this hypothesis [24], we were able to exclude the possibility that participants in blocked mimicry conditions were simply distracted by the mouthguard and did not have the attentional resources necessary to see small differences between smiles. The results of our two experiments provide support for the hypothesis that facial mimicry is used to decode the differences between true and false smiles.

While the previous studies [24], [1], [3], [9], preferentially used pen-in-the-mouth procedures, we asked participants to wear mouthguards in order to limit their facial responses. Our interpretation of the findings is that altered facial mimicry reduces participants' ability to distinguish true and false smiles. Alternatively, however, the use of mouthguard or pen-in-mouth manipulations could prevent participants from generating verbal labels when identifying smiles. Such a disruption of inner speech – rather than blocked facial mimicry – could then be reflected in impaired judgments of smile authenticity. We believe that such an alternative explanation, although consistent with findings from neuroscience linking inner speech with imitation and emotion processing [41], [42], [43], is unlikely in the case of the current studies. First, it is difficult to predict what exactly participants would subvocalize - especially when observing genuine and false smiles – and thus, to anticipate the exact nature and timing of the effects. Secondly, it is possible that the mouthguard and pen do not prevent inner speech because these procedures do not necessarily interfere with inner voice and inner ear (phonological store), critical for subvocalization [44]. Finally and most importantly, if subvocalization underlies emotion recognition, preventing it should disrupt the processing of all facial expressions equally. This is, however, not the case in previous studies that block mimicry: techniques altering the muscles of mouth impair recognition of happiness and disgust, which heavily involve the mouth, but not recognition of fear and anger [3], [9]. Such findings suggest that being able to use facial muscles relevant for a given facial expression may be more essential for recognition than subvocally naming the expression.

Our findings replicate and strengthen the results of Maringer and colleagues [24]. They are also consistent with other evidence implicating embodiment and mimicry in judging the meaning of facial expressions. Namely, Oberman et al. [3] altered facial responses using a variant of the pen-in-the-mouth procedure. Holding the pen with the teeth without touching it with the lips significantly decreased participants' performance, especially when recognizing facial expressions of happiness. Oberman and colleagues' study used static, prototypical expressions of happiness, edited to decrease their intensity. Recognizing such expressions is an arguably difficult task that should recruit embodied simulation processes. However, the forced-choice paradigm asked participants to distinguish between categorically different expressions, such as happiness and disgust (happiness being the only positive emotion), while the current study demonstrated the importance of facial mimicry in making more subtle judgments *within* the category of smiles. This suggests that mimicry does not simply promote emotion category labeling, but also facilitates the detection of fine-grained differences in expression meaning.

More recently, Manera, Grandi, and Colle [45] provided interesting insight into the “embodiment” hypothesis and recognition of subtle facial expressions. The researchers tested participants' accuracy in judging photographs as instances of true and false smiles. Performance varied significantly as a function of participants' tendency to experience emotional contagion. Susceptibility to emotional contagion for *negative emotions*, such as fear, anger, and sadness, predicted more accurate judgments of smile genuineness. But higher levels of susceptibility to emotional contagion for *positive emotions* (happiness, love) predicted lower recognition performance, because such participants categorized most false smiles as sincere. Manera and colleagues [45] did not directly assess or manipulate the facial reactions of the participants. Still, when combined with the current study's demonstration of the role mimicry plays in smile genuineness judgments, it is entirely possible that individual tendencies to simulate the perceived emotion and to produce overt or covert facial mimicry might have been the mechanism underlying differences in participants' judgments. The relationship between emotional contagion and mimicry of non-prototypic facial expressions needs to be explored in further studies.

Despite the growing body of research implicating mimicry in the discrimination between genuine and false smiles, other recent findings suggest that this evidence, although promising, is far from being conclusive. For example, the exact conditions under which spontaneous mimicry improves the recognition of facial expression in general and smile type in particular still need to be examined [10]. Consistently, Korb, With, Niedenthal, Kaiser and Grandjean (2013, unpublished data) presented participants with different types of precisely-manipulated smiles and recorded participants' facial EMG while collecting ratings of smile genuineness. Both smile intensity and participants' facial mimicry predicted judgments of authenticity. Still, Korb and colleagues did not find significant mediation – that is, statistically controlling for participants' facial mimicry did not significantly influence their ratings of smile genuineness. Similarly, a recent study by Slessor, Bailey, Rendell, Huffmann, Henry, and Miles [46] showed that the time course of facial reactions to enjoyment and non-enjoyment smiles differs in young and older adults. More importantly, such differences in facial mimicry did not predict participants' ratings of smile authenticity.

This somewhat complicated literature highlights the need for a better understanding of the effect different types of stimuli, such as static, dynamic, and synthetic, play in judgments of genuineness. Furthermore, a clearer operationalization of smiles would be useful in unraveling these problems. Because the debate about the actual features of “true” and “false” smiles is unresolved, a potential solution is not to create experimental stimuli having these features, but rather to use videos of spontaneously-produced, naturalistic smiles, as we did in the current experiments.

It is also worth noting that in the two EMG studies just described (i.e., Korb et al., 2013, Slessor and colleagues), participants judged authenticity with the electrodes attached to their faces, while in Maringer et al. [24], and in the experiments reported here, genuineness ratings were collected without any invasive measure of mimicry. Moreover, in Maringer's studies and in the present Experiments 2 and 3, facial mimicry was experimentally altered and not measured at its spontaneously occurring levels. On the other hand, studies of Korb et al. (2013) and Slessor and colleagues [46] examined such spontaneous facial mimicry. These and other methodological differences, including the nature of the stimuli used, the action units manipulated, and the experimental design employed do not allow a conclusive explanation of such inconsistent findings. Future studies will need to address the causes of observed discrepancies and attempt to precisely define the conditions under which facial reactions are crucial for correct smile interpretation. Such questions can be explored in constructive replications of existing findings, using different types of smile stimuli, varying experimental designs, and with appropriate control conditions.

Another possible improvement in the investigation of the role of mimicry of smiles is to go beyond the classic distinction of “true” and “false.” Smiles convey a much wider variety of messages, often unrelated to enjoyment per se. Thus, using different types of socially functional smiles and asking participants to judge the extent to which these smiles communicate trustworthiness, embarrassment, or superiority may be more relevant to the situations that participants experience in their daily lives, and offer more possibilities for studying facial mimicry. Future studies in our laboratory will also test new procedures for blocking mimicry of the entire face, including the use of clay or paraffin masks. Another line of research aims to investigate how chronic impairments of facial mimicry in facial palsy patients affect the perception and recognition of facial expressions. A focus of future research will be to investigate whether “mimicry” needs to be observable, involve all of the relevant muscles, and/or be time-locked in order to have functional effects on face processing [47]. Answering such questions has the potential to advance our understanding of how modulations of facial mimicry shape social interactions and group dynamics.

In sum, the present research relied on the strategy of preventing or moderating a supposedly causal mechanism in order to measure predicted changes in performance [5] such as smile discrimination. An important question that the present studies cannot answer is related to the neural mechanisms underlying blocking imitation. Consistently with previous findings from neuroscience, pre-engaging facial musculature with a pen or a mouthguard may alter feedback from face muscles and skin and reduce the subsequent activations of the amygdala as well as the shared representation network involving premotor cortex, inferior frontal gyrus pars opercularis (mirror neuron system), somatosensory cortex, and left anterior insula [48], [49], [50]. The exact alterations in motor outflow induced by mimicry-inhibiting manipulations need to be assessed in further studies. Recent results suggest, however, that these experimental procedures may inhibit the influence of the shared representation network on the motor system [51], [52]. Such preparatory suppression might constitute the mechanism controlling the automatic tendency to imitate.

In the experiments reported here, inhibiting this tendency was related to poorer discrimination of true and false smiles. Our studies not only relate facial mimicry to understanding the meaning of smiles, but they also test novel techniques for manipulating and measuring mimicry. For instance, Experiment 1 in the current paper employs a combination of automatic facial recognition software and EMG recording to correlate the synchrony between the facial expressions of the target and the perceiver. As we develop better tools for manipulating and operationalizing facial mimicry, we will come closer to answering the questions of whether, when, and how mimicry plays a fundamental role in emotion processing.
